# Isotope tracer assessment of exogenous glucose oxidation during aerobic exercise in hypoxia

**DOI:** 10.14814/phy2.14594

**Published:** 2020-09-22

**Authors:** Lee M. Margolis, John P. O’Hara, Alex Griffiths, Robert W. Wolfe, Andrew J. Young, Stefan M. Pasiakos

**Affiliations:** ^1^ U.S. Army Research Institute of Environmental Medicine Natick MA USA; ^2^ Carnegie School of Sport Leeds Beckett University Leeds UK; ^3^ Department of Geriatrics Center for Translational Research in Aging and Longevity Donald W. Reynolds Institute on Aging University of Arkansas for Medical Sciences Little Rock AR USA

1

Our laboratories have consistently shown that unacclimatized lowlanders demonstrate a blunted ability to oxidize exogenous glucose during relative (O'Hara et al., , 2017, 2019) or absolute (Margolis et al., 2019; Young et al., 2018) intensity‐matched aerobic exercise within hours of hypoxia exposure. Our data challenge common recommendations to increase the carbohydrate intake during exercise at high altitude (>2,500 m) to fuel exercise metabolism and augment endurance capability (Koehle, Cheng, & Sporer, 2014). As such, we read with interest the recent report by Sumi et al. (Sumi, Hayashi, Yatsutani, & Goto, 2020), as their findings, on the surface, appear to confirm our previous results (Margolis et al., 2019; O'Hara et al., 2017, 2019; Young et al., 2018). Their randomized crossover study aimed to investigate the effects of acute hypoxia on exogenous glucose oxidation in nine unacclimatized lowlanders performing 30‐min of absolute or relative intensity‐matched aerobic exercise. The investigators’ interpretation of their primary finding was that exogenous glucose oxidation was lower under hypoxic compared to normoxic conditions when exercise was matched for absolute rather than relative intensity.

While these data appear confirmatory, there are methodological limitations in the work by Sumi et al. (2020) that should be acknowledged. Most noteworthy, the only glucose provided by Sumi et al. (2020) was the 0.5 g of oral ^13^C‐glucose isotope tracer before exercise. Surprisingly, no additional glucose (i.e., tracee) was provided. As such, the tracer/tracee (^13^CO_2_/^12^CO_2_) ratios measured by Sumi et al. (2020) reflects the oxidation of a quantitatively trivial amount of exogenous glucose. The article referenced by Sumi et al. (2020) justifying their approach for measuring ^13^C‐excertion after only ingesting the tracer noted that the fasting ^13^C‐glucose breath test was a proposed clinical screening tool designed to reflect the efficiency of hepatic energy utilization (Tanaka et al., 2013). Furthermore, Sumi et al. (2020) do not appear to calculate exogenous glucose oxidation (Peronnet, Rheaume, Lavoie, Hillaire‐Marcel, & Massicotte, 1998). To calculate exogenous glucose oxidation during exercise, the equations of (Mosora et al., 1981) or Peronnet, Massicotte, Brisson, & Hillaire‐Marcel (1990) should be used depending upon the study design:

$$\text{Exogenous Carbohydrate Oxidation}\left(g\cdot \min^{‐1}\right)=\dot{V}{\text{CO}}_{2}\left[\left(R_{\exp}‐R_{\text{ref}}\right)/\left(R_{\text{exo}}‐R_{\text{ref}}\right)\right]/k,$$

where *V̇*CO_2_ is in liters per min, *R*_exp_ is the isotopic composition of expired CO_2_ after isotope consumption, *R*_ref_ is the isotopic composition of expired CO_2_ at rest prior to exercise and isotope ingestion (Mosora et al., 1981) or during exercise with the ingestion of a placebo (Peronnet et al., 1990), Rexo is the isotopic composition of the exogenous glucose ingested, and *k* is a constant for the volume of CO_2_ provided by the complete oxidation of glucose (Peronnet et al., 1998). In one of our previous studies, we calculated exogenous glucose oxidation after giving 0.2 g oral ^13^C‐glucose diluted in water as a placebo versus 0.2 g oral ^13^C‐glucose diluted with 80 g glucose to assess changes in total and exogenous glucose oxidation during acute and chronic hypoxia exposure (Young et al., 2018). The negligible amount of glucose provided in tracer form (i.e., our experimental placebo) expectedly yielded 0 g of oxidized exogenous glucose (Young et al., 2018). As such, the amount of exogenous glucose oxidized by Sumi et al. (2020) should be essentially 0 g. If the authors had used a plasma precursor method they could have distinguished plasma glucose oxidation from total carbohydrate oxidation, with the balance between the two representing glycogen oxidation, but as stated in the manuscript, plasma ^13^C‐glucose enrichments were not measured.

Even if the isotope methodology was carried out correctly, other limitations remain that bring their results into question. When oral ^13^C‐glucose is used to study exogenous glucose oxidation, exercise is typically performed for 80 to 120‐min, and at least the first 40‐min of exercise are not used in the calculation. The exclusion of the initial portion of the exercise is to allow the time for the ^13^C/^12^C in expired CO_2_ to equilibrate with the ^13^C/^12^C produced in tissues. Taking the delay between ^13^CO_2_ production in tissues and at the mouth into account is necessary to ensure the accurate assessment of exogenous glucose oxidation (Peronnet et al., 1998). The 30‐min exercise bout used by Sumi et al. (2020) was likely too short for this equilibration to occur. Such computations have been demonstrated to underestimate exogenous carbohydrate oxidation.

In conclusion, while it appeared that results from Sumi et al. (2020) confirmed previously published findings from our laboratories (Margolis et al., 2019; O'Hara et al., 2017, 2019; Young et al., 2018), careful examination of their methodological approach revealed several limitations that preclude drawing any conclusions regarding the effects of acute hypoxia exposure on exogenous glucose oxidation during aerobic exercise. However, it is clear that exogenous glucose oxidation is lower in unacclimatized lowlanders performing aerobic exercise matched for relative (O'Hara et al., 2017, 2019) or absolute (Margolis et al., 2019; Young et al., 2018) intensities under acute hypoxic conditions compared to normoxia. We are certainly encouraged to see other laboratories reassessing metabolic fueling strategies for exercise at high altitude, as the complex mechanisms contributing to these differences are likely multifactorial, resulting from lower exogenous glucose absorption/release from the gut and impaired peripheral insulin sensitivity and resultant glucose uptake (Margolis et al., 2019). We hope our letter, which serves to highlight the complex intricacies associated with isotopic assessments of human metabolism, provides the fundamental methodological basis for studies to employ when assessing dietary strategies to enhance performance at high altitude.

### DISCLOSURE

The authors declare that they have no conflicts of interest relevant to the content of this article. The opinions or assertions contained herein are the private views of the authors and are not to be construed as official or as reflecting the views of the Army or the Department of Defense.

### Funding information

This material is based on the work supported by U.S. Army Medical Research and Development Command.

REFERENCES

Koehle, M. S.
, 
Cheng, I.
, & 
Sporer, B.
 (2014). Canadian academy of sport and exercise medicine position statement: Athletes at high altitude. Clinical Journal of Sport Medicine, 24, 120–127.2456943010.1097/JSM.0000000000000024

Margolis, L. M.
, 
Wilson, M. A.
, 
Whitney, C. C.
, 
Carrigan, C. T.
, 
Murphy, N. E.
, 
Radcliffe, P. N.
, … 
Pasiakos, S. M.
 (2019). Acute hypoxia reduces exogenous glucose oxidation, glucose turnover, and metabolic clearance rate during steady‐state aerobic exercise. Metabolism, 103, 154030
10.1016/j.metabol.2019.154030
31778707

Mosora, F.
, 
Lacroix, M.
, 
Luyckx, A.
, 
Pallikarakis, N.
, 
Pirnay, F.
, 
Krzentowski, G.
, & 
Lefebvre, P.
 (1981). Glucose oxidation in relation to the size of the oral glucose loading dose. Metabolism, 30, 1143–1149. 10.1016/0026-0495(81)90033-0
6796801

O'Hara, J. P.
, 
Duckworth, L.
, 
Black, A.
, 
Woods, D. R.
, 
Mellor, A.
, 
Boos, C.
, … 
King, R. F.
 (2019). Fuel use during exercise at altitude in women with glucose‐fructose ingestion. Medicine and Science in Sports and Exercise, 51(12), 2586–2594. 10.1249/MSS.0000000000002072
31206498

O'Hara, J. P.
, 
Woods, D. R.
, 
Mellor, A.
, 
Boos, C.
, 
Gallagher, L.
, 
Tsakirides, C.
, … 
King, R. F.
 (2017). A comparison of substrate oxidation during prolonged exercise in men at terrestrial altitude and normobaric normoxia following the coingestion of 13C glucose and 13C fructose. Physiological Reports, 5(1), e13101.2808242810.14814/phy2.13101PMC5256160

Peronnet, F.
, 
Massicotte, D.
, 
Brisson, G.
, & 
Hillaire‐Marcel, C.
 (1990). Use of 13C substrates for metabolic studies in exercise: Methodological considerations. Journal of Applied Physiology, 69(1047–1052), 1990.10.1152/jappl.1990.69.3.10472123176

Peronnet, F.
, 
Rheaume, N.
, 
Lavoie, C.
, 
Hillaire‐Marcel, C.
, & 
Massicotte, D.
 (1998). Oral [13C]glucose oxidation during prolonged exercise after high‐ and low‐carbohydrate diets. Journal of Applied Physiology, 85(723–730), 1998.968875210.1152/jappl.1998.85.2.723

Sumi, D.
, 
Hayashi, N.
, 
Yatsutani, H.
, & 
Goto, K.
 (2020). Exogenous glucose oxidation during endurance exercise in hypoxia. Physiol Rep, 8, e14457
10.14814/phy2.14457
32652803PMC7354086

Tanaka, K.
, 
Matsuura, T.
, 
Shindo, D.
, 
Aida, Y.
, 
Matsumoto, Y.
, 
Nagatsuma, K.
, … 
Suzuki, M.
 (2013). Noninvasive assessment of insulin resistance in the liver using the fasting (13)C‐glucose breath test. Translational Research, 162, 191–200. 10.1016/j.trsl.2013.06.003
23810582

Young, A. J.
, 
Berryman, C. E.
, 
Kenefick, R. W.
, 
Derosier, A. N.
, 
Margolis, L. M.
, 
Wilson, M. A.
, … 
Pasiakos, S. M.
 (2018). Altitude acclimatization alleviates the hypoxia‐induced suppression of exogenous glucose oxidation during steady‐state aerobic exercise. Frontiers in Physiology, 9, 830
10.3389/fphys.2018.00830
30038576PMC6046468
